# Long-Read Haplotype Phasing Resolves Allelic Configuration as a Missing Layer of Precision Oncology

**DOI:** 10.64898/2026.05.05.26351600

**Published:** 2026-05-05

**Authors:** Josh N. Vo, Yi-Mi Wu, Rui Wang, Tiffany Pham, Xuhong Cao, Sophie Yeung, Mingyu Park, Yelena Kleyman-Smith, Guo Ci Teo, Alyssa Wu, Anne Li, Jamie Estill, Lakshmi P. Kunju, Chen Yang, Dan R. Robinson, Arul M. Chinnaiyan

**Affiliations:** 1 Michigan Center for Translational Pathology, University of Michigan Medical School, Ann Arbor, Michigan, USA; 2 Department of Pathology, University of Michigan Medical School, Ann Arbor, Michigan, USA; 3 Rogel Cancer Center, University of Michigan Medical School, Ann Arbor, Michigan, USA; 4 Department of Urology, University of Michigan Medical School, Ann Arbor, Michigan, USA; 5 Howard Hughes Medical Institute, Ann Arbor, Michigan, USA

## Abstract

Conventional short-read sequencing cannot determine whether co-occurring variants within a cancer gene reside on the same allele (*cis*) or on opposing alleles (*trans*), a distinction with direct biological and therapeutic consequences. *Trans* configurations confirm biallelic tumor suppressor inactivation and inform therapy selection, while *cis* configurations generate compound oncogenic alleles with enhanced activity. We analyzed 768 patients with prostate, breast, or ovarian cancers in the PROBLEM cohort, using mutational signatures to nominate cryptic genomic instability cases where the causative biallelic event was not apparent from short-read sequencing. Long-read nanopore sequencing resolved 32 of 46 cryptic cases (69.6%), leveraging its unique advantages in direct methylation detection, long insertion resolution, and complex structural variant characterization, confirming *trans* biallelic inactivation in all resolved tumor suppressor cases. Systematic analysis of 4,496 MiOncoSeq samples identified 17,519 multi-hit gene pairs, of which 78.7% exceeded the 500 bp short-read phasing limit. Long-read phasing further revealed recurrent compound *cis* oncogenic alleles in *NOTCH1*, *PIK3CA*, *PDGFRB*, and *KIT* with functionally synergistic activity. Haplotype phasing resolves a systematically overlooked gap in cancer variant interpretation and warrants broader integration into precision oncology workflows.

## Introduction

The molecular characterization of cancer has undergone significant advancement due to the implementation of second-generation sequencing technologies, commonly referred to as short-read sequencing. This progress has paved the way for precision medicine approaches that align patients with targeted therapies based on the molecular profiles of their tumors.^1,2^ Nevertheless, conventional short-read sequencing platforms possess inherent limitations, particularly in their ability to detect complex structural variants, resolve repetitive genomic regions, and characterize long insertions that may contain clinically actionable information.^3,4^ These diagnostic shortcomings are further exacerbated by the use of targeted gene panels, which, while offering cost-effectiveness and rapid turnaround times, provide limited genomic coverage and resolution.^5^

In contrast, third-generation long-read nanopore sequencing effectively addresses these fundamental limitations. This technology facilitates the detection of complex structural variants and long insertions that are often overlooked by short-read methods, enables the characterization of repetitive elements, and supports haplotype phasing — the experimental resolution of which variants reside on the same versus opposing chromosomal alleles.^3,4,6^ Moreover, nanopore platforms can simultaneously detect epigenetic modifications, such as cytosine methylation, without the need for bisulfite treatment.^6^

Haplotype phasing carries distinct biological and therapeutic consequences depending on the gene context. When two variants co-occur within a cancer gene, their allelic configuration — whether on the same chromosome (*cis*) or on opposing alleles (*trans*) — determines their biological meaning entirely. For tumor suppressor genes, *trans* configuration confirms biallelic inactivation consistent with Knudson’s two-hit model, establishing pathogenicity and eligibility for pathway-targeted therapies such as PARP inhibitors in homologous recombination deficiency (HRD).^7,8^ For oncogenes, *cis* configuration generates compound alleles whose synergistic functional consequences are qualitatively distinct from either variant alone.^9^ Short-read sequencing cannot resolve phase for the majority of clinically relevant co-occurring variant pairs, and computational inference from variant allele frequencies is unreliable in the context of tumor heterogeneity and copy number alterations.^10^

In this study, we systematically evaluated the clinical utility of nanopore sequencing within a cohort of patients with advanced cancers exhibiting suspected genomic instability. First, we ascertain the incremental diagnostic yield of nanopore sequencing in identifying cryptic variants that conventional methods fail to detect and demonstrate that haplotype phasing experimentally confirms *trans* biallelic tumor suppressor inactivation underlying these cryptic genomic instability phenotypes. Second, through systematic analysis of 3,688 patients in the MiOncoSeq clinical sequencing program, we reveal a previously uncharacterized landscape of compound *cis* oncogenic alleles in *NOTCH1*, *PDGFRB*, *KIT*, and *PIK3CA*, with functional evidence of enhanced oncogenic activity and implications for targeted therapy selection.

## Results

### Systematic Identification of Cases with Cryptic Genomic Instability

**Figure 1.**
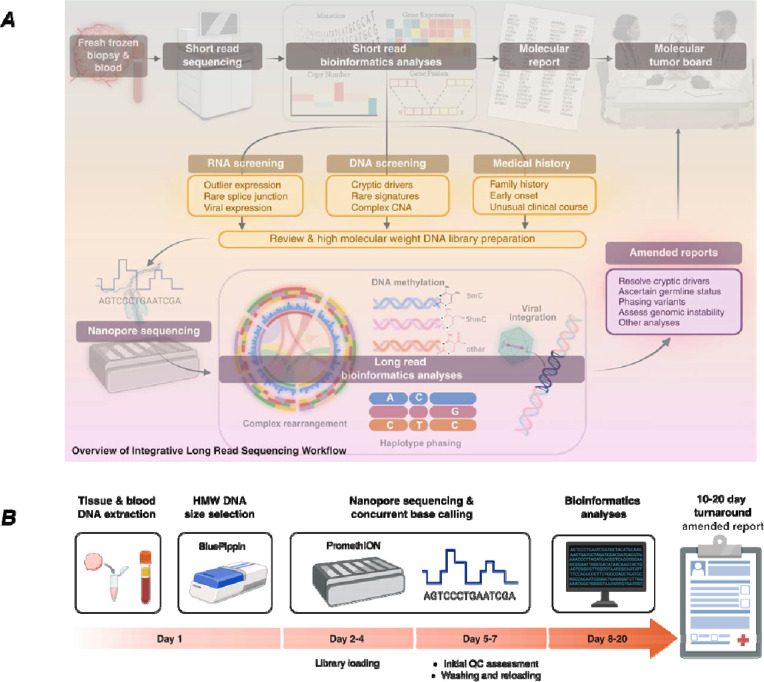
Overview of the integrative long-read sequencing workflow. **(A)** Clinical samples undergo standard short-read whole-exome and targeted panel sequencing as part of routine precision oncology care. Cases are nominated for long-read nanopore sequencing based on three complementary screening criteria: RNA evidence (outlier gene expression, rare splice junctions, viral transcript detection); DNA evidence (cryptic driver candidates, rare mutational signatures, complex copy number architecture); and relevant medical history (family history of cancer, early disease onset, unusual clinical course). Long-read bioinformatics analysis simultaneously characterizes complex structural rearrangements, performs haplotype phasing, and detects DNA methylation directly from base modification signals. Findings are communicated as amended clinical reports to treating oncologists. **(B)** The timeline illustrates the complete workflow from sample receipt to amended report, with a median turnaround of 8–20 days.

We enrolled 768 patients with prostate, breast, or ovarian cancers in the PROBLEM cohort (*Pr*ostate-*O*varian-*B*reast-*L*ikely-*E*lusive-*M*utations) through the MiOncoSeq precision oncology program at the University of Michigan Rogel Cancer Center (Table 1, Methods). All patients underwent short-read whole-exome and targeted panel sequencing as part of routine clinical care, followed by computational mutational signature analysis to identify cases with genomic instability phenotypes. Cases lacking identifiable biallelic drivers despite strong signature evidence were subsequently nominated for long-read nanopore sequencing on the Oxford Nanopore PromethION platform (Figure 1, Methods, Figure S1), contingent upon adequate DNA quality and quantity for library preparation.

**Figure 2.**
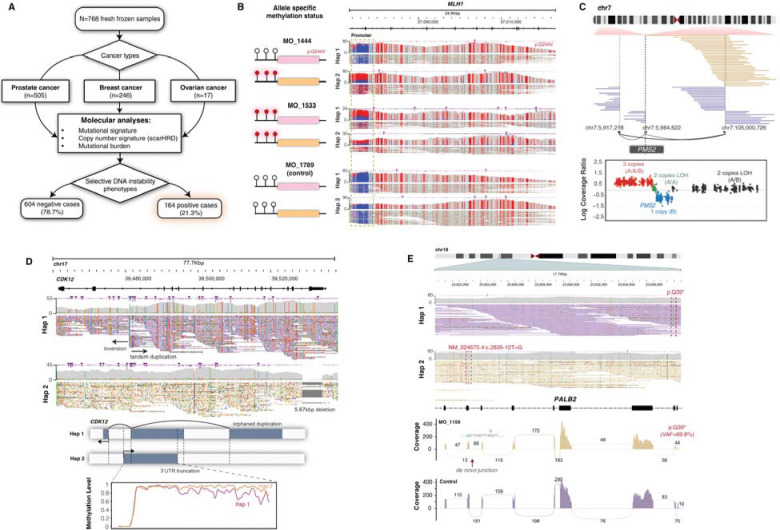
Cryptic mechanisms of genomic instability resolved by long-read sequencing in the PROBLEM cohort. **(A)** Molecular diagnostic workflow for the PROBLEM cohort, showing computational mutational signature-guided nomination of 164 cases with genomic instability phenotypes from 768 patients with prostate, breast, or ovarian cancers. **(B)** Representative cryptic MMRD mechanism: allele-specific methylation tracks across the *MLH1* promoter demonstrating coordinated biallelic hypermethylation in two cases (MO_1444, MO_1533) relative to an unmethylated control (MO_1789). **(C)** Long-read structural variant characterization of a chromothripsis-like *PMS2* rearrangement in a neuroendocrine prostate cancer case (MO_2707). Short-read copy number analysis identified a hemizygous deletion of the *PMS2* region but could not resolve the second hit, which was characterized only by long-read sequencing. **(D)** Representative cryptic FTD mechanism: haplotype phasing enables detection of biallelic *CDK12* inactivation in MO_2674. One allele harbors a 16 kb deletion removing the last exon and 3’ UTR, likely contributing to nonsense-mediated decay. The opposing allele contains an inversion truncating the first 20 kb of *CDK12* and a duplication of the orphaned segment. Haplotype-specific methylation analysis demonstrates progressive loss of epigenetic maintenance across the orphaned copies, with methylation levels declining toward the 3’ region. **(E) Top**: Patient MO_1159 harbors NM_024675.4:c.2835–12T>G, an ultra-rare novel splice *PALB2* intronic variant. SpliceAI^12^ computational prediction demonstrates 94% probability that this germline variant abolishes the canonical splice acceptor site through disruption of the polypyrimidine tract. Haplotype phasing demonstrated that the germline variant occurs in *trans* configuration with a somatic nonsense mutation (p.Gln39Ter), confirming biallelic *PALB2* inactivation. **Bottom**: Validated RNA sequencing analysis demonstrated that this mutation triggers an exon skipping event.

Computational mutational signature analysis identified 164 cases (21.3%) with characteristic patterns of genomic instability (Figure 2A): specifically, HRD (n=106 across prostate, breast, and ovarian cancers), focal tandem duplication (FTD; n=34, predominantly prostate), and mismatch repair deficiency (MMRD; n=24). Cases lacking identifiable biallelic driver events by short-read sequencing despite strong computational evidence for underlying genomic instability were selected for long-read nanopore sequencing.

### Long-Read Sequencing Resolves Cryptic Instability Through *Trans* Phasing

Cryptic MMRD mechanisms encompassed epigenetic and structural alterations, including coordinated *MLH1* promoter hypermethylation with biallelic transcriptional silencing, compound heterozygous *MLH1* methylation coupled with *trans*-acting mutation, and complex structural variants affecting *PMS2* and *MSH2* characterized by chromosomal rearrangements disrupting the remaining functional allele (Figure 2B–C, Table S1). Notably, the complex rearrangement involving *PMS2* in a neuroendocrine prostate cancer patient exhibited chromothripsis-like, copy-neutral characteristics that evaded detection by short-read sequencing (Figure 2C).

FTD cases uniformly involved *CDK12* disruption through previously unrecognized balanced chromosomal rearrangements. Most of these reciprocal translocation events maintained copy number neutrality in one or both allele, rendering them essentially invisible to conventional short-read targeted panel copy number analysis; yet, they were readily characterized through nanopore sequencing’s capacity to traverse complex breakpoint junctions and resolve large-scale structural architecture (Figure 2D, Figure S2A, Table S2).

Among HRD cases, *BRCA1* promoter hypermethylation with concurrent allelic loss emerged as the predominant mechanism in 10 of 20 resolved cases (50%) (Figure S2B, Table S3). Cryptic *BRCA2* inactivation patterns were resolved in 4 cases, characterized by compound heterozygous deletions and germline variant combined with structural rearrangements affecting the *trans* allele (Table S3). Focal *BARD1* deletions encompassing the critical RING domain were detected in 2 cases (one somatic, one germline), representing alterations that evade short-read detection due to the relative paucity of informative single-nucleotide polymorphisms (SNPs) in targeted panels (Figure S3, Table S3). The somatic *BARD1* case harbored deletion of exons 2–3, which would produce isoform β (BARD1β) lacking the RING domain essential for BRCA1 binding, resulting in haploinsufficiency-mediated HRD through reduced functional BRCA1-BARD1 heterodimer formation.^11^ In the germline case, long-read-enabled haplotype phasing revealed an additional cryptic splicing variant of *BARD1* in *trans* with the germline deletion (Figure S3). Additional events include germline structural variants in *BRIP1* and somatic *RAD51C* methylation (Figure S2B, Table S3).

An exemplary case involved a breast cancer patient in her early 50s with an unremarkable family history exhibiting robust computational evidence for HRD yet harboring only a single *PALB2* nonsense variant (p.Gln39Ter) by conventional analysis. Long-read sequencing uncovered an ultra-rare germline deep intronic splice variant positioned 12 base pairs upstream of the canonical splice acceptor site (NM_024675.4:c.2835–12T>G). Computational splice site prediction^12^ indicated the abolishment of normal splicing, which was experimentally validated through RNA sequencing, revealing exon skipping. Haplotype phasing definitively established the *trans* configuration of these variants, confirming biallelic *PALB2* inactivation and pathogenicity of the germline variant (Figure 2E, Table S3).

Initially, nanopore sequencing resolved 32 of 46 cryptic cases (69.6%), with complete diagnostic success for MMRD (5/5, 100%) and FTD (7/7, 100%) (Table S1–3). In all but one *BARD1* case, haplotype phasing was integral to establishing the diagnosis: direct experimental confirmation of the *trans* configuration of two loss-of-function events — one identified by conventional sequencing and one cryptic, transforming a computationally inferred instability phenotype into an experimentally validated biallelic inactivation event.

### Secondary Analyses of Unresolved Cases

**Figure 3.**
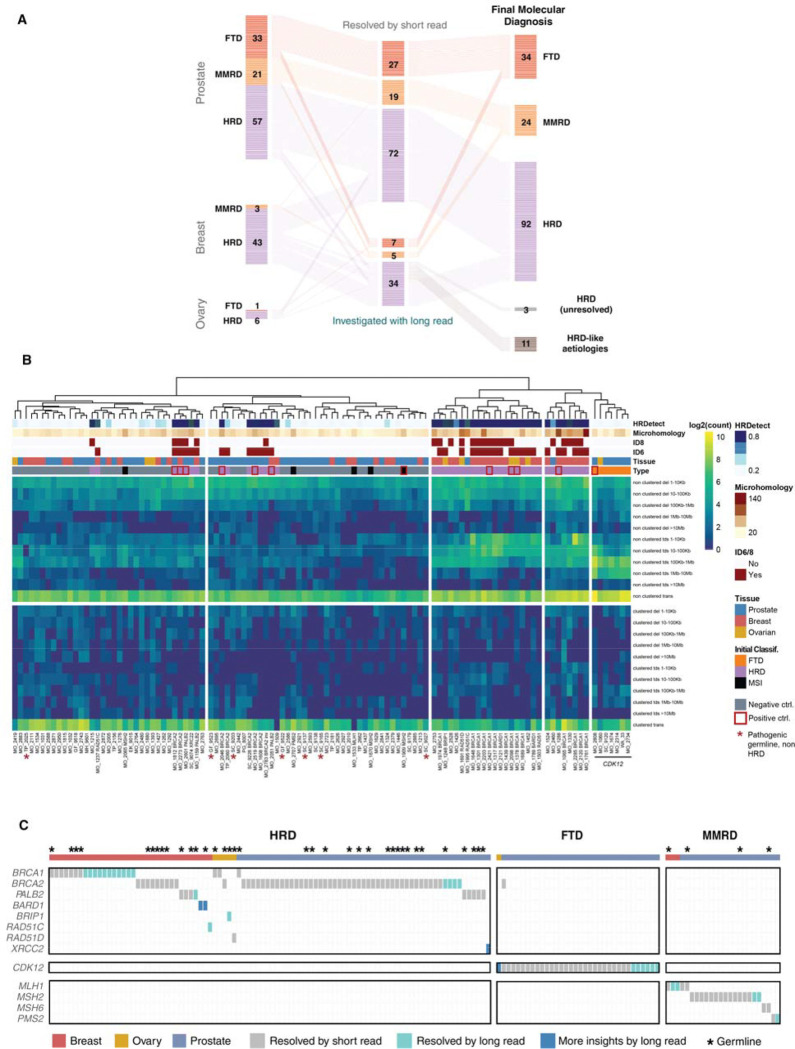
Diagnostic yield and molecular refinement of long-read sequencing in the PROBLEM cohort. **(A)** Alluvial diagram illustrating the flow of diagnostic resolution across MMRD, FTD, and HRD categories in prostate, breast, and ovarian cancers, from initial signature classification through short-read and long-read sequencing, including reclassification of 11 ambiguous HRD cases as HRD-like aetiologies following secondary signature analysis. **(B)** Heatmap displays structural variant features including insertions and deletions stratified by length, extracted using the *SigProfilerExtractor* package. *SigProfilerExtractor* categorizes structural variants as clustered or non-clustered; for this analysis, only non-clustered events were utilized to prevent artificial clustering bias from chromothripsis-associated “clustered” samples. Features underwent log2 transformation to normalize the wide range of structural variant counts and enable comparison across different event types. Manhattan distance calculation was employed because it is more robust to outliers and better suited for count-based data, particularly when analyzing discrete structural variant events. Complete linkage clustering methodology was applied to maximize between-cluster separation and create distinct, well-separated mutational pattern groups. Additional annotation tracks provide complementary mutational signature information: COSMIC indel signatures ID6 and ID8 (classified as positive when contribution ≥20%), quantification of microhomology sequences ≥3bp flanking structural variant breakpoints, and HRDetect scores for homologous recombination deficiency assessment. The analysis incorporates positive control cases with known driver alterations (indicated as red box) identified through short-read sequencing and negative control cases (indicated as gray box in annotation track) definitively lacking genomic instability signatures, providing validation benchmarks to guide interpretation of cryptic mutational mechanisms. Cases with pathogenic germline variants but lacking HRD phenotype were marked with red asterisks. For these analyses, tumor samples without matched normal controls were excluded. All FTD cases clustered together due to their characteristic tandem duplications ranging from 0.1 MB to 10 MB. Overall, breast cancer and ovarian cancer cases with HRD caused by *BRCA1*/*BARD1* alterations also clustered together, reflecting their enrichment for short tandem duplications and short deletions. Some HRD samples with *BRCA2* and *PALB2* deficiency showed less genomic scarring, characterized primarily by enrichment for short deletions. MMRD cases lacked characteristic copy number signatures, consistent with their primary manifestation through point mutations rather than structural alterations. **(C)** Oncoprint of gene-level mechanisms resolved across HRD, FTD, and MMRD cases, distinguishing events resolved by short-read sequencing (gray), resolved by long-read sequencing (teal), and providing additional mechanistic insight through long-read analysis (blue). Asterisks denote pathogenic germline variants.

The initial HRD resolution rate of 58.8% (20/34 cases) left 14 cases unresolved, prompting secondary signature analysis since computational signatures inferred from short-read sequencing may have lower specificity. Comprehensive mutational signature analysis using HRDetect^13,14^ scoring combined with detailed indel and structural variant characterization was performed using methodologies originally developed for short-read whole-genome sequencing but adapted for long-read data (more details in Methods). Specifically, we analyzed COSMIC indel signature 6 (ID6, deletions with microhomology) and indel signature 8 (ID8, deletions at repetitive sequences indicative of defective non-homologous end-joining repair), alongside structural variant signatures SV3 (short tandem duplications between 1–100Kb) and SV5 (short non-clustered deletions between 1–100Kb), which have been shown to be associated with HRD.^13–15^ Integrative analysis revealed that 11 of the 14 unresolved cases exhibited features inconsistent with classical HRD, including low HRDetect^13^ scores, isolated ID8 signatures without accompanying ID6 patterns, or complete absence of both ID6/ID8 and SV3/SV5 signatures despite initial computational HRD classification (Figure 3B and more details in the Methods). Notable examples among these atypical cases included tandem duplication of *RAD51*’s initial exons, potentially altering expression of this essential gene.^16^ We also identified deep deletion of *ERCC4*, a nucleotide excision repair gene^17^ whose loss may force cellular reliance on homologous recombination pathways, overwhelming this repair system and producing HRD-like phenotypes. Only 3 of the 14 cases retained consistent HRD characteristics across all analytical dimensions (mutational patterns, copy number alterations, structural rearrangements, and indel signatures), yielding a *refined* diagnostic accuracy of 20/23 credible HRD cases (86.9%).

Integrative secondary signature analyses also provided additional diagnostic and molecular insights. One prostate cancer patient harbored a homozygous deletion of *XRCC2* (which belongs to the RAD51 paralog complex BCDX2 with RAD51C, RAD51D, and RAD51B)^18^ initially detected by short-read sequencing. Given the rarity of *XRCC2* deficiency in clinical practice, integrative analyses were instrumental to confirm that this patient exhibited features of HRD, potentially benefiting from PARP inhibitor (PARPi) therapy (Figure S4). Conversely, 7 patients harbored known pathogenic germline variants (4 *BRCA2*, 2 *BRCA1*, 1 *BRIP1*) identified through short-read data review. Inspection of the *trans* alleles using long-read sequencing did not reveal additional hits, and comprehensive signature analyses reassured that these cases were non-HRD, indicating they would not benefit from PARPi therapy despite carrying pathogenic germline variants (Figure 3B).

Our phase-aware comprehensive analyses revealed a clinically relevant asymmetry in HRD mechanisms across cancer types. *BRCA1* biallelic inactivation — through mutations, structural variants, copy number, and promoter methylation — was the predominant mechanism in breast cancer (18 of 34 resolved HRD cases) yet was strikingly rare in prostate cancer (1 of 53 resolved HRD cases). This tissue-specific divergence aligns with emerging evidence that BRCA1 may not drive prostate cancer initiation, as reflected by its exceptionally low germline prevalence in prostate cancer cohorts (<1% vs. 8% for *BRCA2*).^19^

Overall, long-read sequencing resolved 32 of 35 credible genomic instability cases (91.4%). When combined with short-read sequencing results, the comprehensive diagnostic yield reached 91.4% for genomically unstable cancers (150 of 164 signature-positive cases) (Figures 3A and 3C). Long-read sequencing thus provided an incremental diagnostic benefit in 4.1% of the entire PROBLEM cohort, with the combined approach identifying actionable findings and molecular insights that influenced treatment decisions in 19.5% of all patients (Figures 3A–C).

Beyond the PROBLEM cohort, long-read sequencing demonstrated its capacity to resolve cryptic germline structural variants that evade standard clinical testing. A woman in her early 60s with a strong family history of breast and ovarian cancer had previously received negative results from commercial multi-gene panel testing and was evaluated outside our institutional cohort. Long-read nanopore sequencing of germline DNA from peripheral blood identified a pathogenic *AluYb8* retrotransposon insertion within exon 25 of *BRCA2* in reverse orientation. The inserted sequence exhibited 99.7% identity to the *AluYb8* consensus, differing by only a single base pair, consistent with a relatively recent insertion event in evolutionary terms.^20^ Target site duplications flanking the insertion were clearly resolved by long reads spanning the full insertion in single molecules, a structural feature invisible to short-read sequencing and absent from standard panel coverage (Figure S5). This case illustrates that long-read germline sequencing is warranted in patients with compelling personal or family histories and uninformative conventional testing, and that the diagnostic gap extends beyond computational instability signatures.

### The Scope of Co-Occurring Variants Requiring Phase Determination

**Figure 4.**
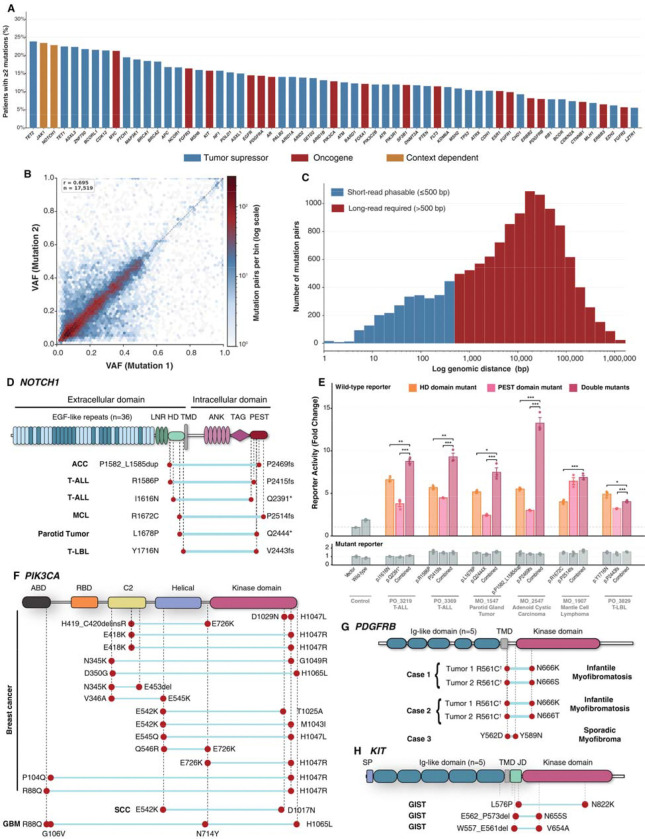
Compound *cis* oncogenic alleles across cancer types revealed by long-read haplotype phasing. **(A)** Frequency of multi-hit events across curated cancer genes in 4,496 tumor samples (3,688 patients). Bars represent the percentage of samples with ≥2 somatic mutations among all samples harboring any mutation in that gene, ordered by decreasing frequency. Blue, tumor suppressor; red, oncogene; amber, context-dependent. **(B)** VAF correlation of co-occurring SNV pairs within the same gene and sample (n = 12,934). Hexagonal bins colored by log-scaled pair count; white bins indicate zero observations. Dashed line, VAF = VAF . Pearson r reported. **(C)** Genomic distance distribution of co-occurring SNV pairs (n = 12,934; log-scaled x-axis). Blue, phasable by short-read sequencing (≤500 bp); red, requiring long-read sequencing (>500 bp). **(D)** NOTCH1 protein schematic showing compound *cis* variant pairs (teal bars) confirmed by long-read phasing across six patients, consistently pairing heterodimerization (HD) domain with PEST domain variants. **(E)** Luciferase reporter assays measuring transcriptional activation of compound NOTCH1 HD+PEST mutations relative to individual variants. Four of six compound pairs showed significantly enhanced NOTCH pathway activation. Unpaired two-tailed Welch’s t-test; n = 3 per group; *p < 0.05, **p < 0.01, ***p < 0.001. **(F)** PIK3CA protein schematic (ABD, RBD, C2, helical, kinase domains) showing compound *cis* variant pairs confirmed by long-read phasing in breast cancer and additional tumor types. Each row represents one patient; circles denote variant positions; dashed lines indicate confirmed *cis* configuration. **(G)** PDGFRB protein schematic showing compound *cis* variants in three mesenchymal tumors: two infantile myofibromatosis cases with germline(†) p.Arg561Cys paired with somatic Asn666 alterations, and one sporadic myofibroma with dual somatic tyrosine substitutions. **(H)** KIT protein schematic showing compound *cis* variants across three cases, spanning juxtamembrane and activation loop domains.

Having established that *trans* phasing is essential for confirming tumor suppressor biallelic inactivation, we asked how broadly the phase ambiguity problem extends across the clinical sequencing landscape. Prior pan-cancer analyses have demonstrated that compound mutations within individual cancer genes are a common phenomenon: among significantly enriched oncogenes, up to 9% of mutated samples harbor co-occurring mutations,^9^ while across all cancer-associated genes, nearly one in four advanced tumors contain composite mutations.^21^ In both studies, oncogene compound mutations were predominantly *cis*-acting, with 78–91% residing on the same allele, yet these estimates relied on RNA sequencing reads or short-read WES data, approaches inherently restricted to variants within a single read length and unable to resolve the majority of clinically relevant pairs.^9,21^ Systematic analysis of 4,496 MiOncoSeq samples across 3,688 patients identified 17,519 co-occurring variant pairs within individual cancer genes, confirming the prevalence of this phenomenon in a clinical sequencing cohort. Multi-hit events were prevalent across both tumor suppressors and oncogenes: among tumor suppressors, *TET2* (23.9%), *NOTCH1* (22.9%), and *ASXL3* (22.4%) showed the highest multi-hit frequencies, while oncogenes including *MYC* (21.3%), *FGFR3* (16.4%), *KIT* (15.8%), *PIK3CA* (12.9%), were also frequently affected (Figure 4A). Whether these co-occurring oncogene variants reside on the same allele, generating hypermorphic configurations with qualitatively distinct oncogenic properties, has remained experimentally unresolvable at clinical scale.

Variant allelic fraction (VAF) correlation analysis restricted to SNV pairs (n=12,934) revealed strong diagonal clustering (r=0.695), a pattern consistent with, but not diagnostic of, *cis* configuration (Figure 4B). This ambiguity is precisely the clinical problem: correlated VAFs can reflect *cis* compound alleles or clonal co-evolution of *trans* variants and cannot be distinguished without experimental phasing. Analysis of genomic distances demonstrated that 78.7% of pairs spanned distances exceeding 500 bp, the practical phasing limit of short-read sequencing, with a median inter-variant distance of 8,420 bp (Figure 4C). Notably, a substantial fraction of pairs exceeded 100,000 bp, driven by mutations in exceptionally large genes such as *LRP1B* (~1.9 Mb) and *NF1* (~280 kb), distances that demand ultra-long nanopore reads for resolution. The overwhelming majority of co-occurring variant pairs in the clinical sequencing landscape therefore require long-read sequencing for definitive allelic assignment. We therefore applied long-read phasing to systematically characterize compound *cis* alleles across recurrently multi-hit oncogenes.

### Compound Cis *NOTCH1* Variants Create Hypermorphic Oncogenic Alleles

NOTCH1 signaling is regulated through a precise autoinhibitory architecture in which the heterodimerization (HD) domain restrains the receptor in a quiescent state until ligand-induced cleavage, while the PEST domain governs intracellular domain degradation.^22^ In our cohort, 22.9% of *NOTCH1*-mutated samples harbored co-occurring variants (Figure 4A), reflecting its context-dependent role as both an oncogene and tumor suppressor across different malignancies.^9,21,22^ Among these, a consistent pattern emerged: a subset of patients harbored one variant in the HD domain paired with one in the PEST domain. Six patients with available high-molecular-weight DNA were selected for long-read sequencing. Long-read sequencing confirmed that, in every case, these variants resided in *cis* on the same allele, across a spectrum of diagnoses including T-cell acute lymphoblastic leukemia, mantle cell lymphoma, adenoid cystic carcinoma, and parotid gland tumor (Figure 4D, Figure S6).

Functional validation using a *NOTCH1* transcription reporter assay demonstrated significantly greater target gene activation in four of six compound HD+PEST double mutants compared to either single mutant alone (*p*<0.001 for significant pairwise comparisons), consistent with synergistic disruption of both autoinhibitory and degradation mechanisms (Figure 4E). The compound mutants showed reporter activity exceeding the additive effects of the individual variants, establishing that *cis* compound *NOTCH1* alleles constitute a qualitatively distinct oncogenic entity. Whether these compound alleles exhibit altered sensitivity to *NOTCH1*-targeted agents or exhibit poorer survival warrants prospective investigation.

### Compound *Cis PIK3CA* Alleles Are Prevalent and Arise in Recurrent Domain-Specific Configurations

*PIK3CA* activating mutations occur in approximately 40% of hormone receptor-positive breast cancers and form the basis for PI3Kα inhibitor therapy with alpelisib, approved in combination with fulvestrant for this indication.^23,24^ The functional consequences of co-occurring *PIK3CA* mutations have been established by complementary pan-cancer studies demonstrating that compound mutations within *PIK3CA* arise more frequently than expected by chance, preferentially in *cis*, and confer enhanced oncogenic activity beyond either variant alone.^9,21,25^ However, the allelic configuration of these variants and therefore their functional and therapeutic interpretation could not be experimentally confirmed by short-read sequencing in prior work.

Among samples with HMW, long-read sequencing identified compound cis *PIK3CA* alleles in 15 breast cancer patients as well as cases spanning brain and squamous cell carcinoma (Figure 4F). Variant pairs encompassed combinations across all five structural domains of p110α — the adaptor-binding domain (ABD), Ras-binding domain (RBD), C2 domain, helical domain, and kinase domain — indicating that compound *PIK3CA* alleles are not restricted to specific hotspot combinations but arise across the full mutational landscape of the gene. The combination spanning the greatest genomic interval in our breast cancer cohort involved p.R88Q in the ABD and p.H1047R in the kinase domain, separated by approximately 35.2 kilobases (chr3:178,916,876 to chr3:178,952,085) (Figure 4F), a distance entirely beyond short-read phasing resolution and confirmed in *cis* only through long-read sequencing.

Two cases beyond breast cancer illustrate the clinical breadth and biological significance of compound *PIK3CA* alleles. A pediatric patient with radiation-induced brainstem glioblastoma harbored four concurrent *PIK3CA* variants confirmed in *cis* by nanopore sequencing: p.R88Q and p.G106V in the ABD, and p.N714Y and p.H1065L in the kinase domain. Despite this extreme allelic complexity, the overall tumor mutation burden was low, indicating that these four variants did not accumulate passively but were each subject to strong positive selection. This pattern is consistent with oncogenic addiction^26^ to a massively hypermorphic *PIK3CA* allele in which progressive intramolecular destabilization across multiple domains drives pathway dependence beyond what any single mutation could achieve.

In contrast, a patient with ocular squamous cell carcinoma, HPV-positive and hypermutated through APOBEC-associated mutagenesis,^27^ harbored three *PIK3CA* mutations: p.E542K in the helical domain, p.R93W in the ABD, and p.D1017N in the kinase domain. Long-read phasing revealed that only p.E542K and p.D1017N resided in *cis*, while p.R93W occupied the opposing allele in *trans*. Critically, all three variants were present at nearly identical variant allele frequencies of approximately 15%, a finding that would conventionally suggest the variants are clonal and indistinguishable in their allelic relationships. This case directly illustrates that VAF is nondiagnostic for phasing in the context of hypermutation and complex clonal architecture, and that only experimental long-read phasing can resolve the true allelic configuration.

### Compound *Cis* Variants in Mesenchymal Tumors

*PDGFRB* and *KIT* are paralogous members of the Type III receptor tyrosine kinase family, sharing five extracellular immunoglobulin-like domains, a juxtamembrane autoinhibitory segment, and a split kinase domain.^28^ Both drive distinct mesenchymal tumor types and are therapeutically targeted by imatinib, making allelic resolution of compound variants in these genes directly actionable.^29,30^

*PDGFRB* kinase activity is regulated through juxtamembrane autoinhibitory interactions involving key residues including Arg561, and germline p.Arg561Cys is the canonical pathogenic variant in familial infantile myofibromatosis.^28,29^ Long-read sequencing confirmed that p.Arg561Cys was in compound *cis* with somatic Asn666 alterations in two independent cases of infantile myofibromatosis, providing the first direct experimental allelic confirmation of the known two-hit mechanism in this disease (Figure 4G, Figure S6). Interestingly, a third sporadic myofibroma case harbored a novel compound somatic configuration: p.Tyr562Asp paired with p.Tyr589Asn in *cis*, representing the first description of dual tyrosine alterations affecting both the juxtamembrane domain and kinase domain N-lobe simultaneously (Figure 4G, Figure S6). While p.Arg561Cys disrupts a single critical autoinhibitory contact, this novel combination potentially destabilizes the juxtamembrane-kinase interface through two independent structural perturbations, raising the possibility of distinct sensitivity to tyrosine kinase inhibitors targeting this regulatory interface.

In gastrointestinal stromal tumor (GIST), primary activating *KIT* juxtamembrane mutations initiate oncogenic signaling, whereas acquired secondary kinase-domain mutations affecting either the ATP-binding pocket or activation loop represent the dominant mechanism of imatinib resistance.^30,31^ Prior mutational analyses suggested that these resistance mutations arise in *cis* with the primary driver allele, but direct experimental confirmation across kilobase-scale genomic intervals separating these variants has remained impractical with short-read sequencing.^32^ Long-read phasing in three patients with documented imatinib progression confirmed *cis* compound *KIT* alleles spanning three mechanistically distinct resistance architectures (Figure 4H): one activation-loop secondary mutation (p.N822K, associated with relative susceptibility to regorafenib-class inhibitors), and two ATP-binding-pocket mutations (the novel p.N655S and p.V654A, the latter classically associated with sunitinib sensitivity).^32^ Phase-aware genotyping in this setting directly informs rational tyrosine kinase inhibitor sequencing because the secondary mutation present on the primary driver allele predicts differential inhibitor susceptibility.

## Discussion

Preliminary attempts have been made in recent years to integrate long-read sequencing into clinical settings, particularly for genetic disorders and select oncological applications.^33–35^ However, these efforts have largely been limited to highly specialized cases, with systematic integration into routine precision oncology workflows remaining largely unexplored. Our study represents one of the first comprehensive evaluations of long-read sequencing in a clinical precision oncology program and uniquely addresses two dimensions that have received insufficient attention: the detection of cryptic pathogenic variants invisible to conventional sequencing and the experimental resolution of variant phase across clinically relevant genomic distances.

While targeted short-read sequencing resolved 71.9% of cases with genomic instability signatures, it left 28.0% mechanistically unexplained despite strong computational evidence. Long-read sequencing resolved 69.6% of these previously cryptic cases, achieving complete resolution for all focal tandem duplications and mismatch repair deficiency cases, and resolving the majority of credible homologous recombination deficiency cases. The combined diagnostic approach yielded actionable findings that influenced treatment decisions in 19.5% of all patients studied. These findings support a **tiered** diagnostic algorithm that deploys long-read sequencing in contexts where it adds maximal value: tumors with strong genomic instability signatures but no identifiable biallelic driver; patients with compelling family histories or multiple primaries despite negative panel results; and tumors with atypical molecular or phenotypic features such as unexplained gene overexpression or paradoxical clinical behavior. As platform costs decline and analytical pipelines mature, we anticipate progressive movement of long-read sequencing toward earlier implementation.

Beyond resolving co-occurring variants with known mechanisms, long-read sequencing uniquely detects pathogenic events that are structurally invisible to short-read methods — including complex balanced rearrangements maintaining copy number neutrality, deep intronic splice variants beyond panel coverage, and mobile element insertions such as the *AluYb8* retrotransposon identified in our germline *BRCA2* case. Crucially, methylation is detected simultaneously from the same nanopore run without bisulfite treatment, enabling direct epigenetic characterization of promoter silencing and allele-specific imprinting in the same assay that performs variant calling and haplotype phasing.

The haplotype phasing capabilities of long-read sequencing translate directly into clinical decision-making across a range of scenarios that are common in precision oncology practice. For instance, when a patient with a mismatch repair deficiency signature initially lacks identifiable biallelic drivers but subsequently demonstrates biallelic somatic *MLH1* promoter methylation through nanopore sequencing, this finding substantially reduces the likelihood of an additional cryptic germline variant, minimizing the burden and urgency of extensive family screening. Conversely, when a germline variant of uncertain significance co-occurs with a strong genomic instability signature, demonstrating its *trans* configuration with a somatic inactivation event through haplotype phasing provides strong evidence for pathogenicity, directly informing clinical management and family counseling decisions. Our *PALB2* case illustrates this precisely: a cryptic deep intronic splice variant on the opposing allele would have remained entirely undetected by conventional sequencing, and its pathogenicity would never have been established without long-read detection and *trans* phase confirmation.

Short-read sequencing, and particularly targeted panel sequencing, may create clinical dilemmas in the setting of apparently monoallelic tumor suppressor inactivation. A patient with a single detected *BRCA1* mutation or hemizygous deletion and an HRD-suggestive signature presents an interpretive challenge: is this true biallelic inactivation with a cryptic second hit, or a phenocopy driven by an alternative mechanism? Long-read sequencing addresses both questions simultaneously — structural variant characterization, copy number analysis, methylation profiling, and mutational signature assessment from a single assay, combined with haplotype phasing to assess the *trans* allele comprehensively. The converse scenario is equally important: a patient with a known germline variant in *BRCA1*, *BRCA2*, *PALB2*, *BARD1*, *RAD51C* or *BRIP1* who develops cancer — even at an early age — should not be automatically assumed to have functional HRD unless supported by strong computational signature evidence or complete resolution of the *trans* allele. Germline carrier status alone is insufficient; biallelic inactivation must be confirmed, and long-read phasing provides the experimental evidence to do so.

In the case of oncogenes, compound *cis* alleles in *EGFR*, *NOTCH1*, *PIK3CA*, *PDGFRB*, and *KIT* warrant systematic investigation to enable genotype-based patient stratification. It has long been proposed that tumors develop increased dependency on individual signaling pathways through oncogene addiction.^26^ The progressive accumulation of compound *cis* variants within a single oncogene represents a molecular substrate for this phenomenon, pointing to pathway-specific therapeutic vulnerabilities that may be invisible to gene-level mutation calls alone. Distinct compound allele combinations likely confer characteristic therapeutic responses and clinical outcomes not captured by single-variant classification.

A critical and underappreciated consequence of phase-agnostic clinical practice is that most trials enrolling patients on the basis of gene mutation status are blind to allelic configuration. As a result, trials may inadvertently mix monoallelic carriers and true compound mutant cases within the same enrollment arm, diluting effect sizes, obscuring genotype-response relationships, and potentially misattributing resistance or exceptional responses to the wrong molecular substrate. Prospective stratification by confirmed allelic phase is now technically feasible and should be incorporated into trial design going forward.

Current clinical genomics reporting frameworks do not routinely operationalize variant phase or epigenetic allele status as structured report elements. The Association for Molecular Pathology / American Society of Clinical Oncology / College of American Pathologists somatic variant interpretation guidelines emphasize genomic coordinates, transcript accession, variant allele fraction, and tier classification, but do not include allelic phase as a required field.^36^ The European Society for Medical Oncology 2024^37^ clinical genomic reporting recommendations likewise address major genomic biomarkers without defining how co-occurring variants within the same gene should be phased in clinical reports. By contrast, Human Genome Variation Society nomenclature formally supports phase annotation through bracket syntax: c.[2376G>C;3103del] denotes *cis* configuration, whereas c.[2376G>C];[3103del] denotes *trans*, yet this notation is rarely used in oncology reporting.

We propose that phase annotation and allele-specific methylation status be incorporated whenever multiple variants occur within the same clinically relevant cancer gene. A practical framework, analogous to existing tiered reporting logic, could distinguish **Level I**, experimentally confirmed phase by long-read sequencing or another long-range method; **Level II**, high-confidence inferred phase based on VAF concordance or linkage evidence; and **Level III**, unresolved phase explicitly reported as unknown. Long-read phase determination may be particularly warranted when two variants co-occur in HRD-, FTD-, or MMRD-associated genes with strong mutational signature evidence, in oncogenes with known compound-allele biology such as *NOTCH1*, *PIK3CA*, *KIT*, or *PDGFRB*, or when a variant of uncertain significance accompanies a known pathogenic variant in a tumor suppressor gene. Incorporating these fields would extend existing report tables and convert currently latent allelic information into clinically interpretable annotations.

## Methods

### Study Design and Patient Cohorts

This study undertook a retrospective analysis of patients who participated in the MiOncoSeq precision oncology program at the University of Michigan Rogel Cancer Center between January 2010 and December 2024. Patient samples, including fresh-frozen tumor tissue, matched blood, and buccal DNA, were collected following written informed consent under protocols approved by the University of Michigan Institutional Review Board (Michigan Oncology Sequencing Protocol, MiOncoSeq and PEDS_MiOncoSeq; IRB HUM00046018, HUM00067928, HUM00056496). All clinical sequencing was performed within our Clinical Laboratory Improvement Amendments (CLIA)-certified molecular diagnostics laboratory. The PROBLEM cohort (Prostate-Ovarian-Breast-Likely-Elusive-Mutations) comprised patients with advanced prostate cancer (n=505), breast cancer (n=246), and ovarian cancer (n=17) who had accessible frozen tumor tissue with greater than 30% tumor purity confirmed by pathological review. Detailed demographics and cancer characteristics of the PROBLEM cohort are provided in Table 1. The broader MiOncoSeq cohort (n=4,496)^38–40^ encompassing all enrolled cancer types was analyzed for systematic characterization of co-occurring variant pairs.

### Integrative Clinical Sequencing by the Illumina Platform

Tumor genomic DNA and total RNA were extracted from fresh-frozen tissue using the AllPrep DNA/RNA/miRNA Kit (Qiagen). Matched normal genomic DNA was isolated from peripheral blood or buccal swab samples using the DNeasy Blood & Tissue Kit (Qiagen). For nanopore sequencing, DNA samples were size selected using Ampure XP beads in a buffer containing 0.75M NaCl and 20% PEG8000 to remove fragments smaller than 2 kb before library preparation. All cancer patients included in this study had previously undergone short-read sequencing on the Illumina platform. This included whole-exome sequencing, targeted exon sequencing, and/or capture-based RNA sequencing, as previously described.^38–40^

### Long-Read Sequencing Protocols

The size distribution of input genomic DNA was assessed using pulsed-field gel electrophoresis (Pippin Pulse; Sage Science). Samples with a high proportion of short DNA fragments were further processed using the BluePippin High-Pass DNA Size Selection System (Sage Science) to enrich for fragments greater than 5 kb, suitable for nanopore sequencing.

Sequencing libraries were prepared using the Ligation Sequencing Kit V14 (SQK-LSK114; Oxford Nanopore Technologies) in combination with the NEBNext Companion Module for Oxford Nanopore Technologies Ligation Sequencing (E7180S; New England Biolabs), following the manufacturers’ protocols. Briefly, 1 μg of high–molecular–weight (HMW) genomic DNA underwent end-repair, A-tailing, and adaptor ligation. Final libraries were purified using AMPure XP beads, quantified with a Qubit 3.0 Fluorometer (Thermo Fisher Scientific), and sequenced on the PromethION P24 platform equipped with A100 flow cells (Oxford Nanopore Technologies).

### NOTCH1 Reporter Assay

NOTCH1 reporter assays were conducted to assess the functional impact of individual and combined *NOTCH1* mutations. Constructs encoding single or double *NOTCH1* mutations were generated and cloned into mammalian expression vectors. 293FT cells were co-transfected with the *NOTCH1* mutant constructs, a NOTCH1-responsive reporter plasmid (RBP-Jk Reporter; SABiosciences/Qiagen), and a Renilla luciferase control plasmid (pRL-TK) using FuGENE HD (Promega), following the manufacturer’s protocol.

Forty-eight hours post-transfection, luciferase activity was measured using a dual-luciferase reporter assay system (Promega). Firefly luciferase activity, driven by the *NOTCH1*-responsive promoter, was normalized to Renilla luciferase activity to control for transfection efficiency. Fold activation of NOTCH signaling was calculated relative to cells transfected with empty vectors, enabling direct comparison of pathway activity across different mutant constructs.

### Short-Read Sequencing Bioinformatics Analyses

Whole-exome and targeted panel sequencing were performed on Illumina HiSeq and NovaSeq 6000 platforms, generating paired-end reads of 100–150 bp with average library fragment sizes of 350 bp. Raw sequencing reads underwent quality control using *FastQC*.^41^ Alignment to the human reference genome (GRCh37/hg19) was performed using *NovoAlign* (v4.02.02)^42^ with default parameters optimized for clinical sequencing. Post-alignment processing included duplicate marking and sorting using *NovoSort*^42^, followed by recalibration of base quality scores and local realignment around indels using GATK best practices.^43^

Somatic and germline variant calling employed a multi-caller approach utilizing *FreeBayes* (v1.3.6)^44^ for single-nucleotide variants and small indels, and *Pindel* (v0.2.5 b8)^45^ for structural variants and larger indels. Copy number alterations were detected using *cnatools*^38–40^ that jointly estimate tumor purity and ploidy. For RNA sequencing, reads were aligned using *STAR* (v2.7.10a)^46^ with two-pass mode, and gene fusion detection was performed using *Arriba* (v2.4.0)^47^ and *STAR-Fusion* (v1.15.0)^48^ with stringent filtering criteria to minimize false positives. Ancestry (Table 1) was inferred from genotype data using *PLINK* (v1.9)^49^ for quality control and linkage disequilibrium pruning, followed by *ADMIXTURE* (v1.3)^50^ for model-based clustering analysis with reference populations from the 1000 Genomes Project.^51^

### Long-Read Sequencing Bioinformatics Analyses

Sequencing was performed on PromethION flow cells (R10.4.1 chemistry) with real-time basecalling using *Dorado*^52^ high-accuracy (hac) mode (Model: dna_r10.4.1_e8.2_400bps_hac@v5.0.0). Quality control assessment was conducted using *ToulligQC* (v2.7.1)^53^, which provided comprehensive metrics, including read length distribution, quality scores, and throughput analysis. Our long-read DNA preparation protocol yielded N50 (the length at which 50% of assembled sequences are longer than this value) values averaging 12,388 bp (median: 10,598 bp, range: 5,137–75,004 bp across samples), ensuring adequate read lengths for structural variant detection and haplotype phasing. Coverage depth analysis revealed average tumor coverage of 83.7X (range: 32.8X-149.8X) and average normal coverage of 40.1X (range: 13.1X-68.9X), providing sufficient depth for haplotype phasing and calling single nucleotide variants (SNVs), insertions/deletions (indels), and structural variants (SVs), including subclonal variants.

Read alignment to GRCh38 was performed using *minimap2* (v2.28)^54^ with long-read specific parameters (-ax map-ont). Variant calling employed *Clair3* (v1.0.11)^55^ for germline variants and *ClairS* (v0.4.1)^56^ for somatic variants, both optimized for R10.4.1 chemistry (model r1041_e82_400bps_hac_v420). Variant filtering and normalization were performed with *bcftools* (v1.20).^57^ Haplotype phasing was conducted using *WhatsHap* (v2.7)^58^ on matched normal samples using high-quality SNVs only, then this phasing information was applied to tumor BAM files.

Structural variant detection was performed with *Severus* (v1.5)^59^, and initial calls were filtered against a curated database of recurrent technical artifacts observed in our cohort (by collapsing breakpoints to a window of 100bp) and a list of established noisy regions.^60^ Copy number analyses on long-read samples were adapted from the methodology of *cnatools*^38–40^. Briefly, zygosity data calculated from single nucleotide polymorphism (SNP) variant allelic fraction (VAF) and log-coverage-ratio (tumor/normal) was segmented using circular binary segmentation algorithm. Ploidy and purity were jointly estimated by expectation-maximization (EM) algorithm. Methylation analysis was enabled during basecalling for 5mC and 5hmC detection (5mCG_5hmC model), with downstream analysis using *modkit*^61^ (v0.4.4) for differential methylation calling and allele-specific methylation analysis. Variant annotation was carried out with *snpEff*^62^, *VEP*^63^, and *SpliceAI*.^12^ The entire pipeline was automated and implemented on the Google Cloud Platform, utilizing scalable computing resources to ensure completion within clinical timelines (Figure S1).

### Initial Mutational Signature Analyses to Nominate Patients With Genomic Instability From Short-Read Sequencing

Since patients from the PROBLEM cohort were sequenced using whole-exome sequencing and targeted panels^38–40^, we focused on mutational signatures applicable to these platforms rather than those requiring whole-genome sequencing (WGS). Single nucleotide signature analysis was performed using *SigProfilerAssignment* (v0.2.2)^64^ with COSMIC v2 reference signatures in 96 trinucleotide contexts.^65^ Homologous recombination deficiency (HRD) assessment was further supplemented by R package *scarHRD*^66^. This approach uses short-read-derived copy number and zygosity data to calculate composite scores from HRD-LOH (loss of heterozygosity), large-scale transitions (LST), and telomeric allelic imbalances (TAI).^67^ In addition to COSMIC signatures, mismatch repair deficiency (MMRD) status was assessed using *MSISensor* (v0.6).^68^

Overall, we used the following criteria to nominate if a patient possessed HRD, MMRD, or focal tandem duplication (FTD) phenotypes:

- MMRD inclusion criteria required either: (1) COSMIC v2 signatures 6, 15, 20, or 26 (if any of these signatures contributed ≥20%)^69^ with tumor mutation burden >10 mutations/Mb, or (2) MSISensor score >10%.
- HRD classification required: (1) scarHRD composite score ≥60 (slightly higher than the standard ≥42 threshold to account for the lower resolution of targeted sequencing^70^), or (2) combined contribution of COSMIC v2 signatures 3 and 8 ^13^ ≥30% with tumor mutation burden ≥2.5 mutations/Mb.
- FTD phenotype was defined by ≥20 focal tandem duplications (0.3–3Mb) inferred from short-read copy number data.^71^

### Secondary Mutational Signature Analyses Post-Nanopore Sequencing

Since nanopore sequencing completely resolved MMRD and FTD cohorts (Figure 3B), secondary mutational signature analyses were mainly focused on HRD. Unlike WES and targeted panels where signature analyses are restricted to mutational and copy number signatures, post-nanopore sequencing enables utilization of indels and structural variants. Specifically, we screened for the presence of ID6 signature (deletions with microhomology), ID8 signature (deletions at repetitive sequences), structural variant signature SV3 (short tandem duplications between 1–100Kb), and SV5 (short non-clustered deletions between 1–100Kb), which have been associated with HRD. ^15,72^ Indel and structural variant signature assignments were performed with *SigProfilerExtractor*^73^ and *SigProfilerAssignment*.^64^ We also applied the well-established HRDetect algorithm^13^ implemented in the R package *signature.tools.lib*.^14^

Based on literature reviews and clinical practices, credible HRD cases required: (1) HRDetect score >0.7 with presence of ID6 signature or SV3/SV5 signatures^15,72^, (2) HRDetect scores 0.5–0.7 with (cryptic) double hits identified by long-read sequencing, or (3) exclusion of cases with isolated ID8 signatures and HRDetect scores < 0.7 as likely representing alternative DNA repair deficiencies or other etiologies.^72,74^ Overall, this analysis identified that only 3 of 14 unresolved cases were credibly HRD (Figure 3B).

### Data Visualization

Genomic data inspection and visualization were performed using IGV (v2.19.1)^75^ and JBrowse^76^ (v3.6.3) for genome browsing and alignment visualization. Methylation analysis utilized Methylartist (v1.5.0)^77^ for allele-specific methylation plotting. Structural variant visualization employed Ribbon (v2.0)^78^ for chromosomal rearrangement plots. Statistical analysis and data visualization used R packages including ggplot2 (v3.5.2)^79^, ComplexHeatmap^80^ (v2.13.1), and pheatmap^81^ (v1.0.12). Scientific illustrations were created using BioRender (biorender.com).

## Supplementary Material

Supplement 1

## Figures and Tables

**Table 1.** Patient Characteristics in the PROBLEM Cohort.

Characteristic	Breast Cancer (n=246)	Prostate Cancer (n=505)	Ovarian Cancer (n=17)
**Cancer Subtype**			
Ductal	212 (86.2%)	—	—
Lobular	24 (9.8%)	—	—
Other	10 (4.1%)	—	—
mCRPC	—	354 (70.1%)	—
Hormone naive	—	126 (25.0%)	—
Neuroendocrine	—	25 (5.0%)	—
Serous	—	—	14 (82.3%)
Non-serous	—	—	3 (17.6%)
**Sex**			
Male	2 (0.8%)	505 (100.0%)	0 (0.0%)
Female	244 (99.2%)	0 (0.0%)	17 (100.0%)
**Median age — yr (range)**	56 (21–83)	68 (36–93)	62 (28–77)
**Race — no. (%)**			
White	221 (89.8%)	435 (86.1%)	15 (88.2%)
Black	20 (8.1%)	53 (10.5%)	1 (5.9%)
Asian	4 (1.6%)	12 (2.4%)	1 (5.9%)
Other	1 (0.5%)	5 (1.0%)	0 (0.0%)
**Receptor status — no. (%)**			
ER-positive, HER2-negative	117 (47.6%)	—	—
HER2-positive	23 (9.3%)	—	—
Triple negative	106 (43.1%)	—	—

## Data Availability

Deidentified nanopore sequencing data supporting the conclusions of this study are available in the database of Genotypes and Phenotypes (dbGaP) under accession number phs000673. Novel pathogenic germline variants identified in this study are being submitted to ClinVar. Additional supporting data are available from the corresponding authors upon reasonable request.

## Statement of Significance

Short-read sequencing cannot resolve whether co-occurring variants within a cancer gene are *cis* or *trans*, a distinction critical for clinical interpretation. Long-read nanopore sequencing addresses this gap through direct haplotype phasing, methylation detection, and complex structural variant resolution, confirming biallelic tumor suppressor inactivation and revealing compound *cis* oncogenic alleles with enhanced activity.
