# Supplementary material for: Long-Read Haplotype Phasing Resolves Allelic Configuration as a Missing Layer of Precision Oncology
Source: medRxiv. 2026 May 5:2026.05.05.26351600. Preprint. [Version 1] doi: 10.64898/2026.05.05.26351600 (PMC13174754; doi:10.64898/2026.05.05.26351600)
Supplement: Supplement 1 [file media-1.docx]

***Supplementary Appendix to:***

**Long-Read Haplotype Phasing Resolves Allelic Configuration as a Missing Layer of Precision Oncology**

Josh N. Vo, Ph.D.^*,1,2,3^, Yi-Mi Wu, Ph.D.^*,1,2,3^, Rui Wang, M.S.^1,2,3^, Tiffany Pham, M.P.H.^1,2,3^, Xuhong Cao, M.S.^1,2,3^, Sophie Yeung^1,2,3^, Mingyu Park^1,2,3^, Yelena Kleyman-Smith^1,2,3^,

Guo Ci Teo, Ph.D.^1,2,3^, Alyssa Wu, B.S.^1^, Anne Li, B.S.^1^, Jamie Estill, Ph.D.^1,2,3^,

Lakshmi P. Kunju, M.D.^2^, Chen Yang, Ph.D.^2^,

Dan R. Robinson, Ph.D.^†,1,2,3^, and Arul M. Chinnaiyan, M.D., Ph.D.^†,1,2,3,4,5^

^1^ Michigan Center for Translational Pathology, University of Michigan Medical School, Ann Arbor, Michigan, USA

^2^ Department of Pathology, University of Michigan Medical School, Ann Arbor, Michigan, USA

^3^ Rogel Cancer Center, University of Michigan Medical School, Ann Arbor, Michigan, USA

^4^ Department of Urology, University of Michigan Medical School, Ann Arbor, Michigan, USA

^5^ Howard Hughes Medical Institute, Ann Arbor, Michigan, USA

* Co-first authors

† Co-corresponding authors: danrobi@med.umich.edu; arul@med.umich.edu

**Running title:** Variant Phase as a Missing Layer of Precision Oncology

**Declaration of interests:** The authors declare no competing interests.


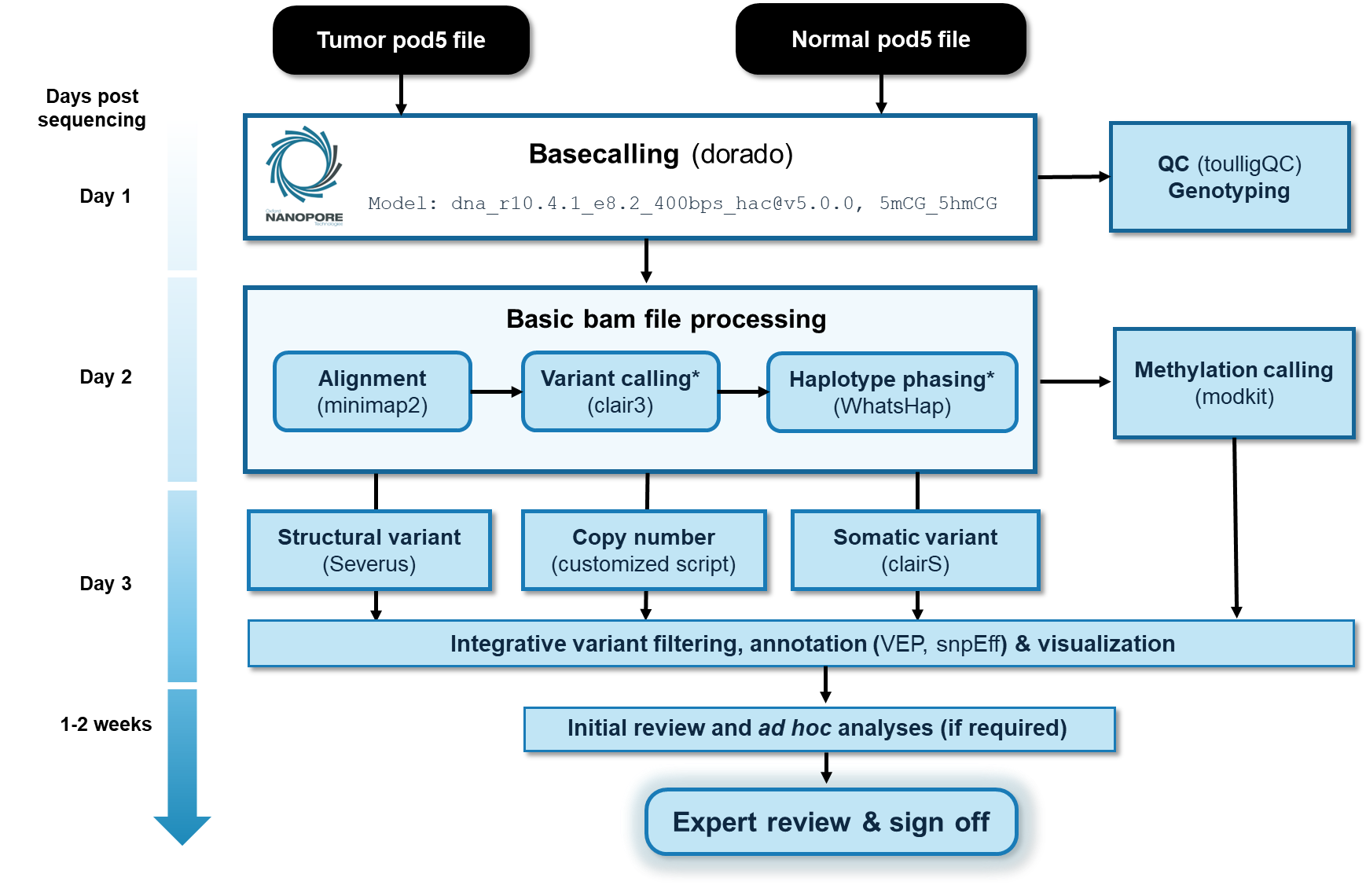


**Figure S1. Long-read bioinformatics pipeline.**

Comprehensive bioinformatics workflow for clinical long-read sequencing analysis. The pipeline encompasses high-accuracy model-based base calling using Dorado (Oxford Nanopore), quality control assessment, read alignment to GRCh38 using minimap2, variant calling with Clair3/ClairS, structural variant detection using Severus, haplotype phasing with WhatsHap, and methylation analysis with modkit for 5mC/5hmC detection (**Methods**). Tasks marked with asterisks are performed only on normal samples, unless the analysis is tumor-only.


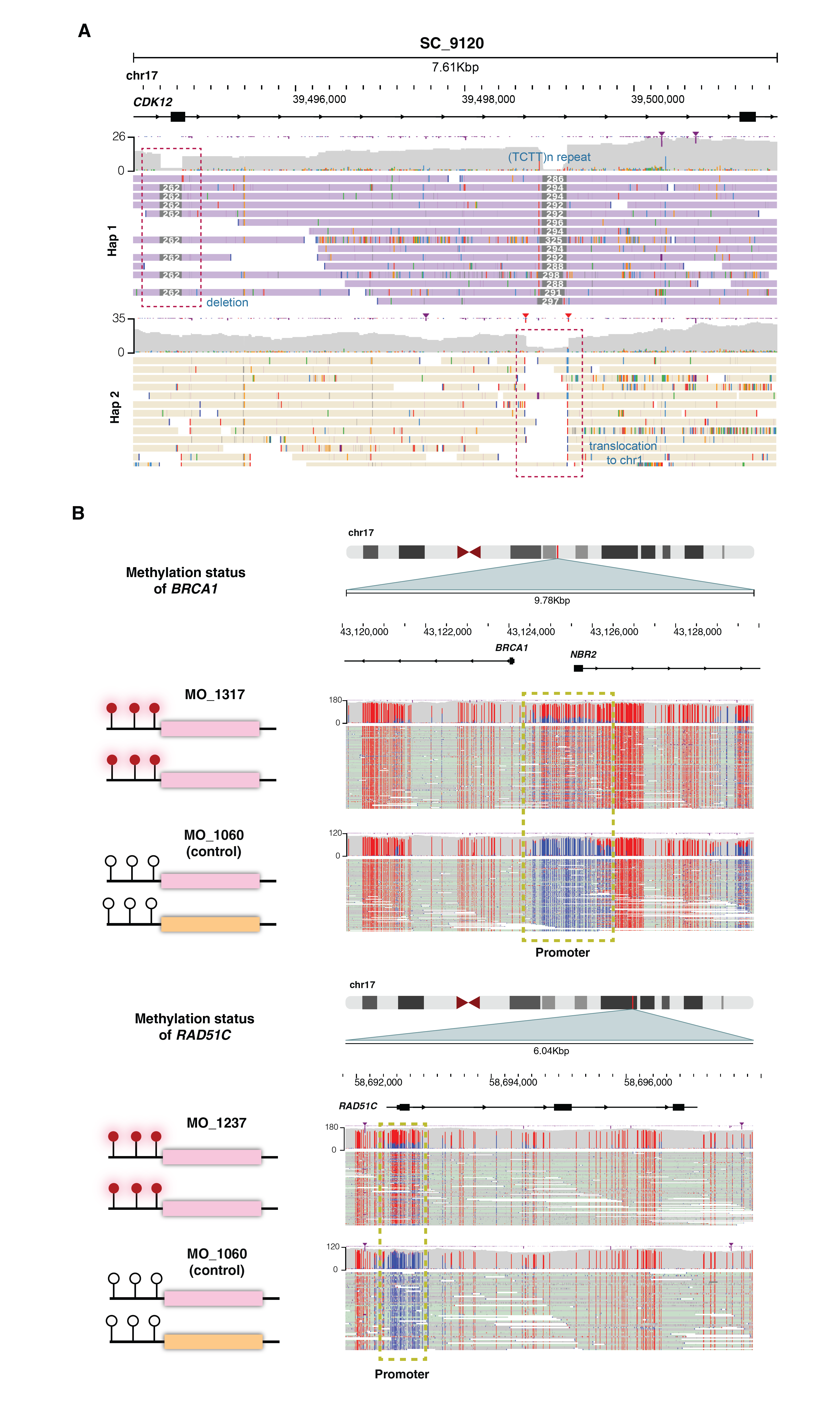


**Figure S2. Additional cryptic FTD and HRD mechanisms.**

**(A)** JBrowse visualization showing biallelic *CDK12* inactivation in case SC_9120. One allele contains a 262bp deletion partially disrupting exon 5; haplotype phasing revealed a reciprocal copy-neutral translocation to chromosome 1 on the opposing allele.

**(B)** Jbrowse screenshots show examples of promoter methylation affecting *BRCA1* and *RAD51C*.


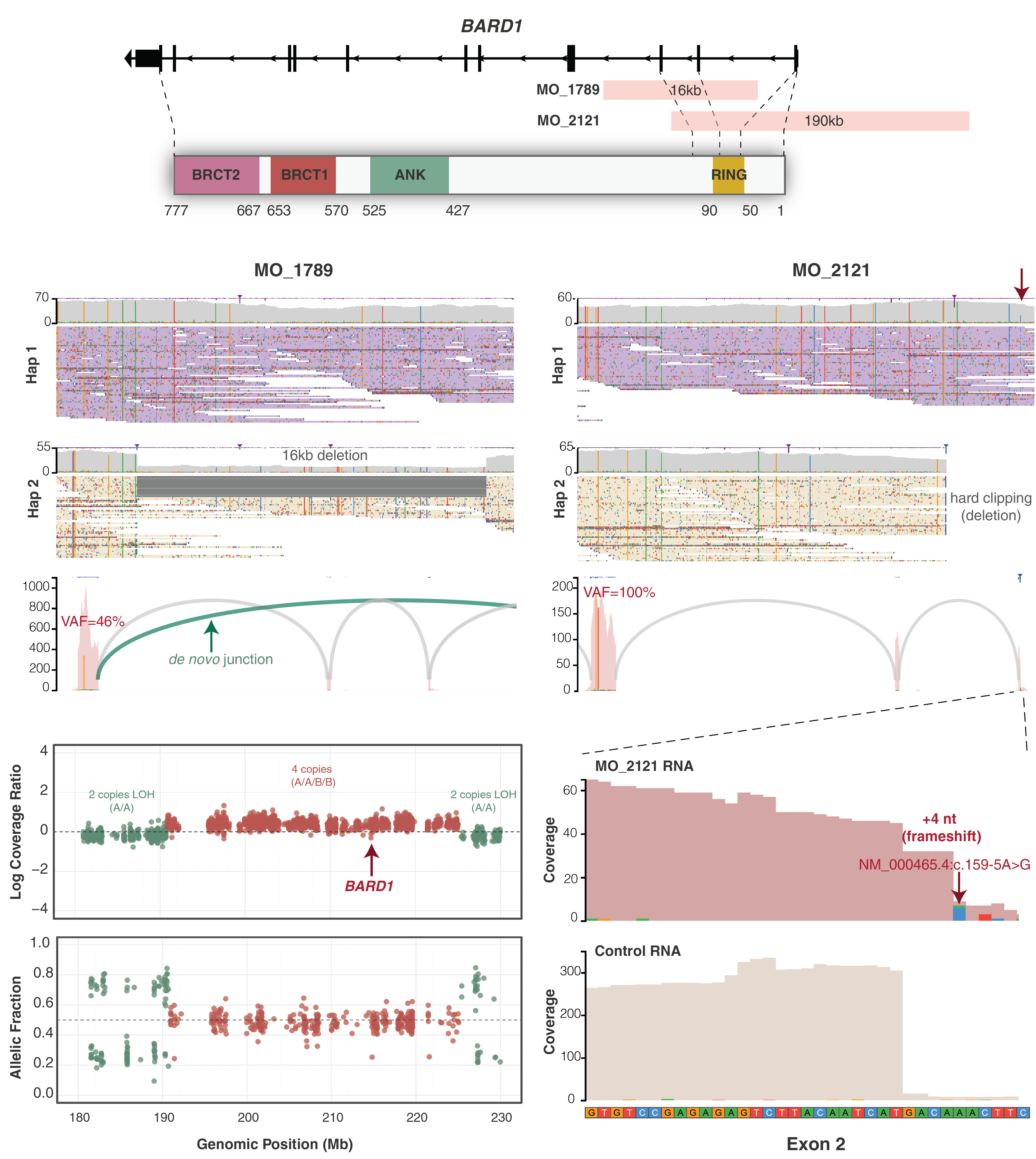


### **Figure S3. Cryptic *BARD1* variants in HRD patients.**

### **Top**: Gene diagram outlines two deletions affecting the RING domain of *BARD1* near the N-terminus. **Bottom**: Detailed genomic analysis. **Left**: The shorter deletion (16kb) is somatic, involving exons 2 and 3. This creates a *de novo* junction that corresponds to isoform beta (*BARD1*-β) that has been suggested to possess oncogenic activities. Interestingly, while this deletion is easily visible in JBrowse visualization of long-read sequencing, it escaped short-read sequencing detection due to two reasons: 1) the breakpoints are located in deep intronic regions, and 2) the paucity of SNPs in such a focal region evaded the segmentation methods used to detect copy number changes (bottom panel). **Right**: The longer deletion is germline (190kb), removing exon 1, exon 2, and the promoter. Integrative haplotype and allele-specific gene expression analysis demonstrated that expression derived entirely from the wild-type allele, ruling out alternative transcription initiation sites for the truncated *BARD1* variant. Careful inspection of the other allele revealed a somatic intronic variant upstream of the exon 2 splice acceptor (NM_000465.4:c.159-5A>G) that creates a new splice acceptor, shifting the reading frame by 4bp. Thus, *BARD1* variants are biallelic in this case.

**
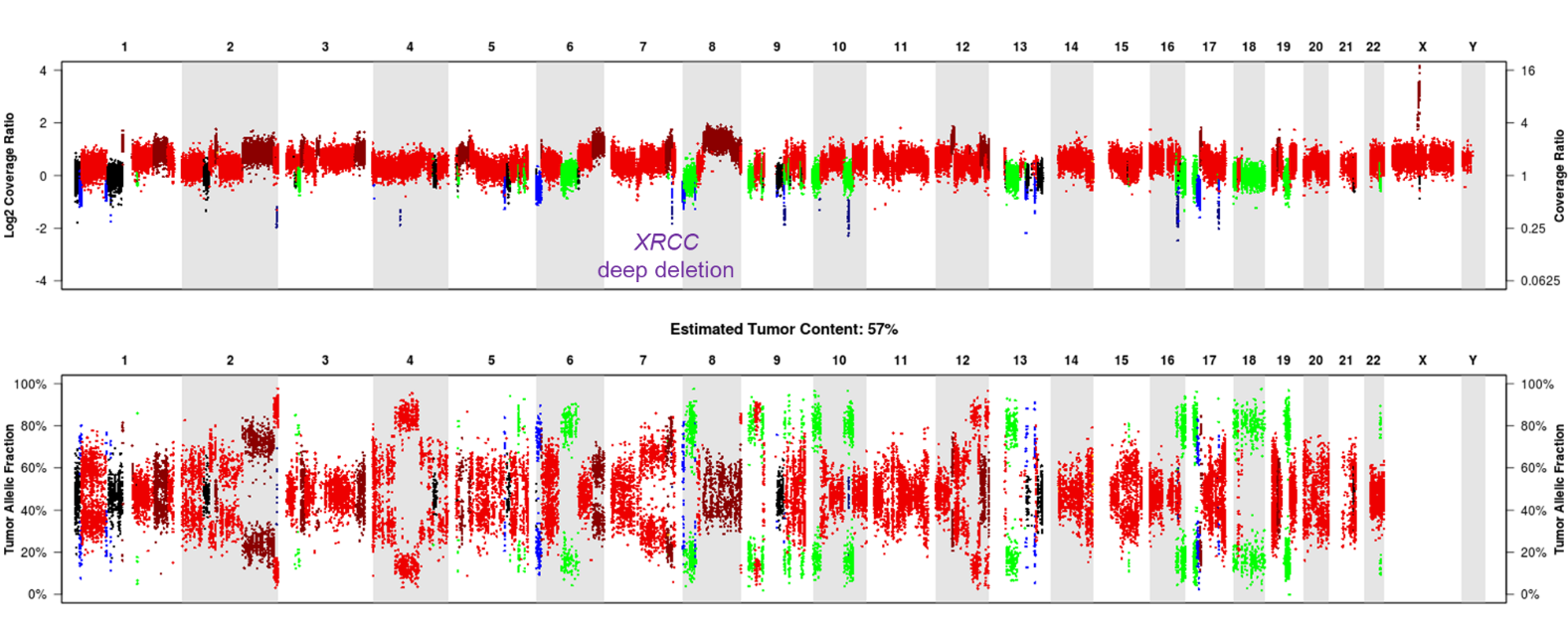
**

**Figure S4. Long-read sequencing resolves cryptic homologous recombination deficiency through XRCC2 deep deletion.**

A prostate cancer patient harboring an elevated scarHRD score lacked an identifiable causative variant, leaving HRD status unconfirmed in the initial clinical report. Long-read nanopore sequencing resolved this cryptic case by independently corroborating HRD-associated mutational signatures and identifying a deep deletion encompassing *XRCC2*, a core homologous recombination repair gene, as the underlying driver of genomic instability.

**
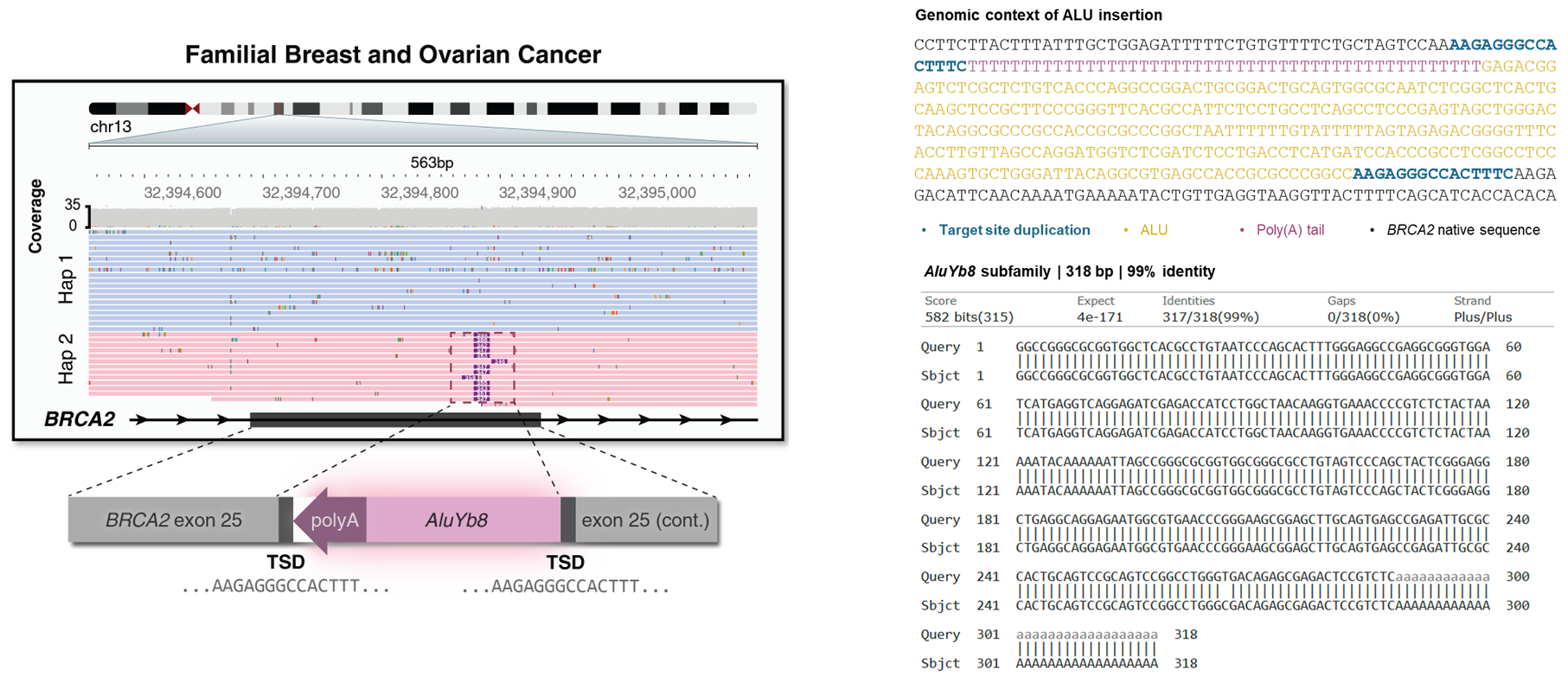
**

**Figure S5. *AluYb8* retrotransposon insertion in *BRCA2*.**

Genomic sequence analysis details the structural features of a pathogenic *AluYb8* insertion within *BRCA2* exon 25, detected in a patient with a strong family history of breast and ovarian cancer and prior negative commercial multi-gene panel testing. Target site duplications and insertion boundaries are shown. BLAST alignment results demonstrate 99.7% sequence identity to the *AluYb8* consensus sequence, differing by only a single nucleotide, consistent with a relatively recent insertion event. Long-read sequencing enabled unambiguous detection of this insertion, which is invisible to standard short-read sequencing.

**
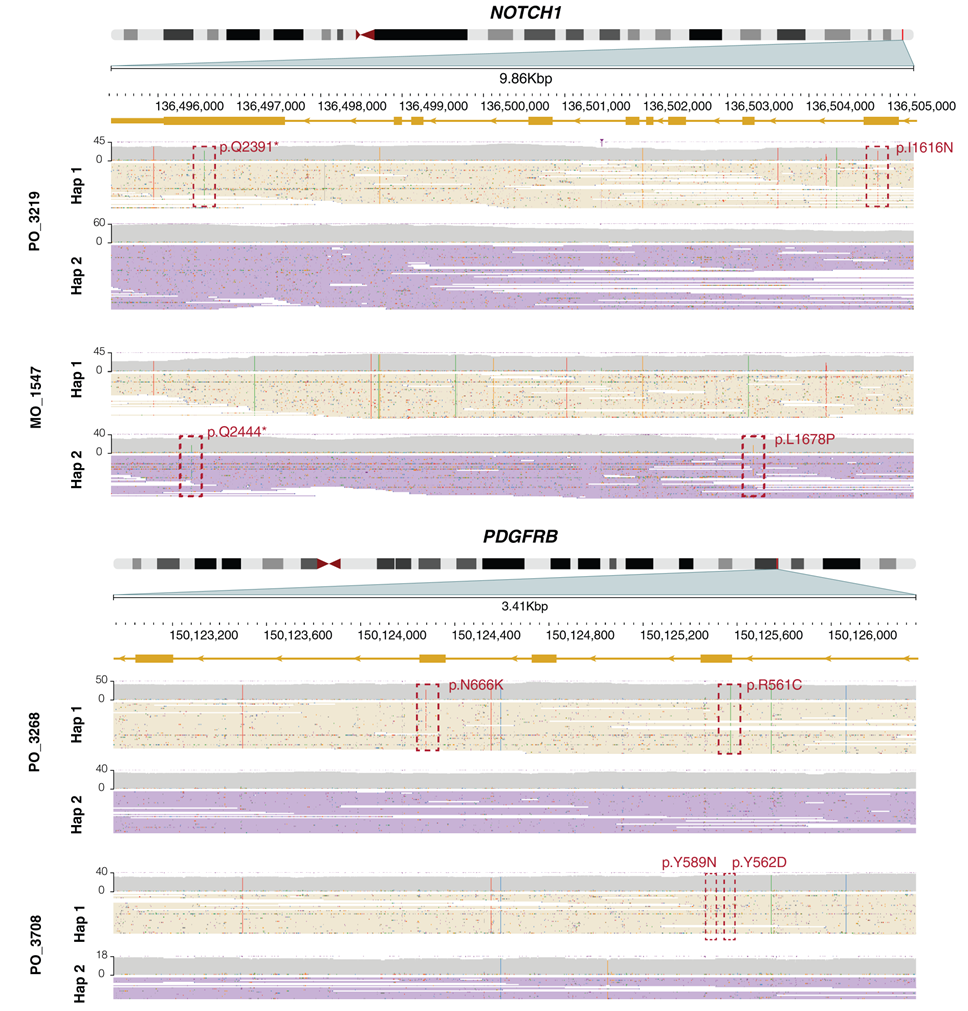
**

**Figure S6. JBrowse** **screenshots confirming *cis* configuration of compound *NOTCH1* and *PDGFRB* variants.**

JBrowse visualization demonstrates definitive *cis* configuration of compound variants in *NOTCH1* (affecting heterodimerization and PEST domains) and *PDGFRB* (affecting juxtamembrane regulatory regions). Long-read sequencing enables unambiguous determination of variant phasing across kilobase-scale distances that are irresolvable by short-read methods. Individual long reads spanning both variant positions are shown for representative cases from each gene.

**Supplementary Tables**

| **Table S1. Resolved Mismatch Repair Deficiency (MMRD) Cases with Cryptic Double Hits.** | | | |
| --- | --- | --- | --- |
| **Patient** | **Cancer Type** | **First Hit** | **Second Hit** |
| MO_2065 | Prostate cancer (mCRPC) | *MSH2* deletion | *MSH2* inversion |
| MO_1670 | Prostate cancer (mCRPC) | *MSH2* deletion | *MSH2* translocation |
| MO_2707 | Neuroendocrine prostate cancer | *PMS2* deletion | *PMS2* complex rearrangement |
| MO_1444 | Breast cancer | *MLH1* promoter methylation | *MLH1* p.Gly244Val (rs63750303)§ |
| MO_1533 | Breast cancer | *MLH1* promoter methylation | *MLH1* promoter methylation |

§ Variant identified by short-read sequencing

| **Table S2. Resolved Focal Tandem Duplication (FTD) Cases with Cryptic Double Hits.** | | | |
| --- | --- | --- | --- |
| **Patient** | **Cancer Type** | **First Hit** | **Second Hit** |
| MO_1674 | Prostate cancer (mCRPC) | *CDK12* tandem duplication | *CDK12* translocation |
| MO_2314 | Prostate cancer (mCRPC) | *CDK12* focal deep deletion | LOH |
| MO_2674 | Prostate cancer (mCRPC) | *CDK12* complex rearrangement | *CDK12* 3’UTR deletion |
| MO_2734 | Prostate cancer (mCRPC) | *CDK12* inversion | LOH |
| SC_9120 | Prostate cancer (mCRPC) | *CDK12* exon 5 partial deletion | *CDK12* translocation |
| WA_33 | Prostate cancer (mCRPC) | *CDK12* translocation | *CDK12* translocation |
| MO_1060 | Serous ovarian cancer | *CDK12* alternative exon usage | LOH |

| **Table S3. Resolved Homologous Recombination Deficiency (HRD) Cases with Cryptic Drivers.** | | | |
| --- | --- | --- | --- |
| **Patient** | **Cancer** | **First Hit** | **Second Hit** |
| MO_1065 | Breast cancer | *BRCA1* methylation | *BRCA1* methylation |
| MO_1301 | Breast cancer | *BRCA1* methylation | *BRCA1* methylation |
| MO_1317 | Breast cancer | *BRCA1* methylation | *BRCA1* methylation |
| MO_1439 | Breast cancer | *BRCA1* methylation | *BRCA1* methylation |
| MO_1689 | Breast cancer | *BRCA1* methylation | *BRCA1* methylation |
| MO_1761 | Breast cancer | *BRCA1* methylation | *BRCA1* methylation |
| MO_2120 | Breast cancer | *BRCA1* methylation | *BRCA1* methylation |
| MO_2285 | Breast cancer | *BRCA1* methylation | *BRCA1* methylation |
| TP_2239 | Breast cancer | *BRCA1* methylation | *BRCA1* methylation |
| MO_1648 | Breast cancer | *BRCA1* methylation | *BRCA1* methylation |
| MO_2203 | Breast cancer | *BRCA1* internal tandem dup | LOH |
| MO_1789 | Breast cancer | *BARD1* partial deletion | - |
| MO_2121 | Breast cancer | *BARD1* partial deletion† | *BARD1* (NM_000465.4:c.159-5A>G) |
| MO_1159 | Breast cancer | *PALB2* p.Gln39Ter§ | *PALB2* (NM_024675.4:c.2835-12T>G)† |
| MO_1237 | Breast cancer | *RAD51C* methylation | LOH |
| MO_1248 | Ovarian cancer | *BRIP1* internal deletion† | LOH |
| MO_1608 | Prostate cancer | *BRCA2* inversion | *BRCA2* deletion |
| MO_2783 | Prostate cancer | *BRCA2* inversion | *BRCA2* p.Thr2968fs †§ |
| SC_9226 | Prostate cancer | *BRCA2* internal deletion | *LOH* |
| TP_2060 | Prostate cancer | *BRCA2* complex SV | *LOH* |

†Germline variants

§Variants identified by short-read sequencing
